# Supplementary material for: Prognostic Factors for Mortality, Activity of Daily Living, and Quality of Life in Taiwanese Older Patients within 1 Year Following Hip Fracture Surgery
Source: J Pers Med. 2022 Jan 13;12(1):102. doi: 10.3390/jpm12010102 (PMC8778381; doi:10.3390/jpm12010102)
Supplement: Supplementary file 1 [file jpm-12-00102-s001.zip › jpm-1496209-supplementary.pdf]

Supplementary Materials

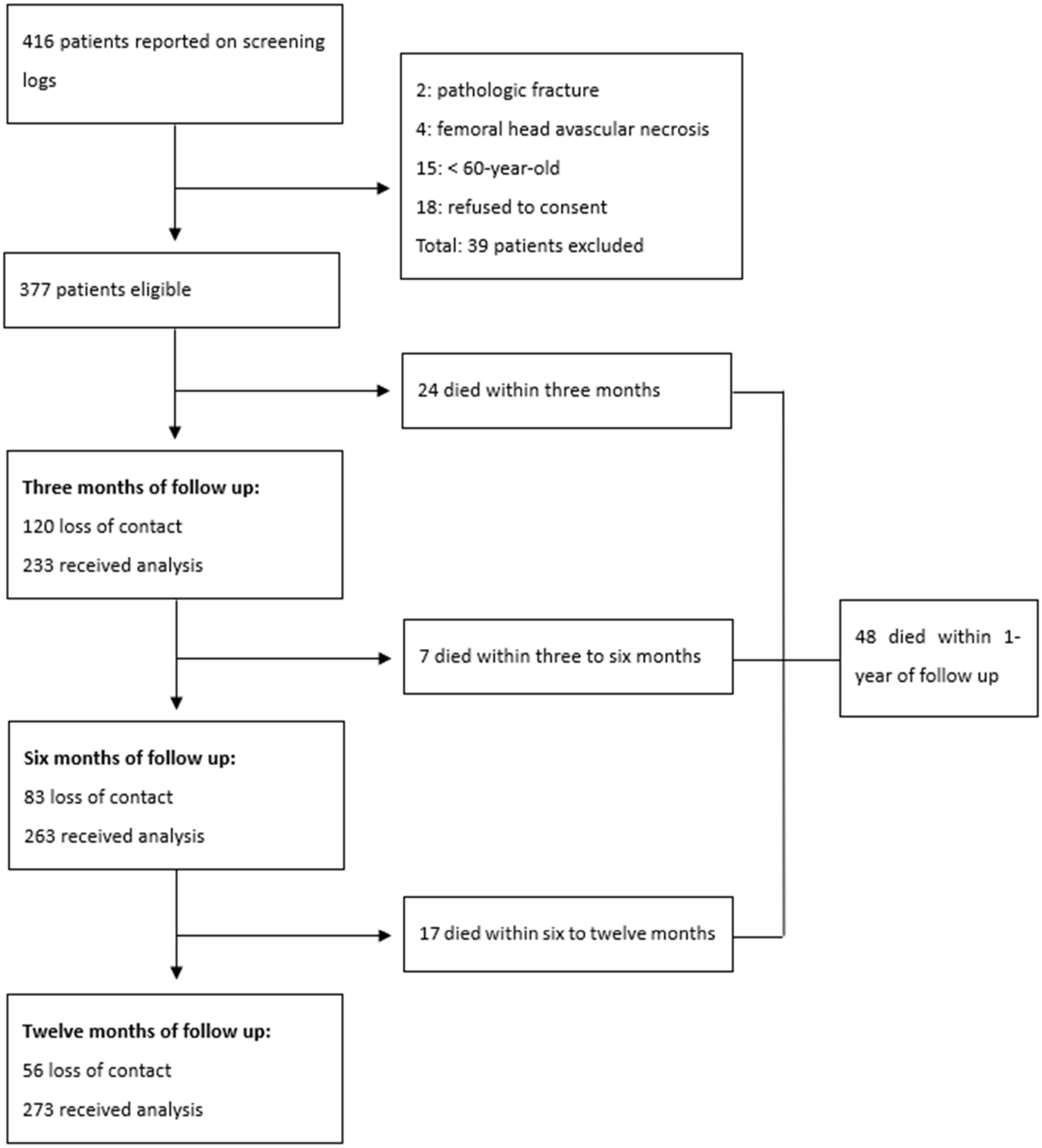

Figure S1. Study profile.

Table S1: Univariate analysis of potential clinical risk factors for postoperative 1-year mortality in older adults with hip fracture.

**Clinical Characteristics (*n* = 377)**	**Survival Group (*n* = 329)**	**Mortality Group (*n* = 48)**	***p*-value**
**Age**	80 ± 9.3	87 ± 9.2	**<0.001**
**Gender**			
Male	88 (84%)	17 (16%)	0.2
Female	241 (89%)	31 (11%)	
**BMI**	22 ± 3.6	21 ± 3.9	0.07
**CCI**	4.7 ± 1.6	6.0 ± 2.4	**<0.001**
**Types of fracture**			
FNF	182 (90%)	21 (10%)	0.1
PTF	147 (85%)	27 (15%)	
**ASA grading**	2.6 ± 0.5	3.0 ± 0.3	**<0.001**
**SPMSQ score**	3.2 ± 3.6	5.8 ± 3.8	**<0.001**
**Handgrip strength (kg)**	13 ± 9.8	8.0 ± 4.1	**<0.001**
BMD T-score	− 3.8 ± 1.2	− 4.0 ± 0.83	0.17
**Pre-operation laboratory data**			
Hb (g/dl)	12 ± 2.2	11 ± 2.4	**0.001**
Na (mmol/L)	137 ± 4.1	137 ± 3.9	1.0
Creatinine (ml/min/1.73m^2)	1.1 ± 1.2	1.5 ± 1.6	0.07
Vitamin D3 (ng/mL)	20 ± 16	20 ± 13	0.9
Parathyroid hormone (pg/mL)	55 ± 65	59 ± 36	0.7
Albumin (g/dL)	3.1 ± 0.4	3.0 ± 0.4	**0.022**
**Surgical record**			
Surgical delay (hour)	76 ± 245	89 ± 142	0.7
Operation time (min)	80 ± 42	70 ± 31	0.1
Blood loss (cc)	113 ± 113	97 ± 73	0.4
**Surgical classification**			
ORIF	204 (86%)	32 (14%)	0.5
Joint replacement	125 (89%)	16 (11%)	

Abbreviations: ASA, American Society of Anesthesiologists; BMD, bone mineral density; BMI, body mass index; CCI, Charlson Comorbidity Index; Hb, hemoglobin; ORIF, open reduction and internal fixation; SD, standard deviation; SPMSQ, Short Portable Mental Status Questionnaire.

Table S2. Univariate analysis of GEE model with multiple imputation: potential risk factors affecting postoperative EQ-5D in older adults with hip fracture.

Variables	β	*p*-value	95% CI Ratio	
			Lower	Upper
		**EQ-5D**		
**Postoperative 3 months v.s. preinjury status**	**–0.12**	**< 0.001**	**–0.14**	**− 0.10**
**Postoperative 6 months v.s. preinjury status**	**–0.08**	**< 0.001**	**− 0.11**	**− 0.05**
**Postoperative 12 months v.s. preinjury status**	**− 0.08**	**< 0.001**	**− 0.11**	**− 0.05**
**Age**	**− 0.007**	**< 0.001**	**− 0.01**	**− 0.005**
Gender (Female vs. Male)	− 0.002	0.92	− 0.04	0.04
BMI	0.003	0.28	–0.002	0.01
**CCI**	**− 0.034**	**< 0.001**	**− 0.04**	**− 0.03**
**BMD T-score**	**0.04**	**< 0.001**	**0.03**	**0.06**
**Pre-operation Hb** (g/dl)	**0.016**	**< 0.001**	**0.007**	**0.02**
Creatinine (mg/dL)	− 0.006	0.42	− 0.02	0.008
Na (mmol/L)	0.002	0.27	− 0.002	0.007
Vitamin D (ng/mL)	0.0002	0.82	–0.001	0.001
PTH	< –0.001	0.22	− 0.001	0.0001
**Albumin** (g/dL)	**0.14**	**< 0.001**	**0.09**	**0.19**
**SPMSQ**	**–0.026**	**< 0.001**	**− 0.03**	**− 0.02**
**ASA grading**	**− 0.12**	**< 0.001**	**− 0.15**	**− 0.09**
**Handgrip strength** (kg)	**0.007**	**< 0.001**	**0.005**	**0.009**
**Types of fracture** (PTF vs. FNF)	**–0.05**	**0.003**	**− 0.09**	**− 0.02**
Surgical delay (hour)	< –0.001	0.94	− 0.0001	0.0001
Surgical classification (Joint replacement vs. ORIF)	< 0.001	0.99	− 0.04	0.04
Time (min)	< –0.001	0.97	− 0.0004	0.0004
Blood loss (cc)	< –0.001	0.56	− 0.0002	0.0001

Abbreviations: ASA, American Society of Anesthesiologists; BMD, bone mineral density; BMI, body mass index; CCI, Charlson Comorbidity Index; FNF, femoral neck fracture; Hb, hemoglobin; PTF, pertrochanteric fracture; ORIF, open reduction and internal fixation; SD, standard deviation.

Table S3: Univariate analysis of the GEE model with multiple imputation: factors affecting postoperative BI in older adults with hip fracture.

Variables	β	*p*-value	95% CI Ratio	
			Lower	Upper
		**BI**		
**Postoperative 3 months v.s. preinjury status**	**− 16.5**	**< 0.001**	**− 18.7**	**− 14.3**
**Postoperative 6 months v.s. preinjury status**	**− 13.9**	**< 0.001**	**− 16.6**	**− 11.1**
**Postoperative 12 months v.s. preinjury status**	**− 14.3**	**< 0.001**	**–17.5**	**− 11.2**
**Age**	**− 0.7**	**< 0.001**	**− 0.98**	**− 0.46**
Gender (Female vs. Male)	− 0.84	0.77	− 6.5	4.8
BMI	0.57	0.10	− 0.12	1.3
**CCI**	**− 4.5**	**< 0.001**	**− 5.9**	**− 3.2**
**BMD T-score**	**4.5**	**< 0.001**	**2.3**	**6.8**
**Pre-operation Hb** (g/dl)	**1.9**	**0.001**	**0.77**	**3.06**
Creatinine (mg/dL)	− 0.34	0.74	− 2.4	1.7
Na (mmol/L)	0.37	0.24	− 0.24	1.0
Vitamin D (ng/mL)	0.009	0.93	− 0.18	0.19
PTH	− 0.03	0.13	− 0.08	0.01
**Albumin** (g/dL)	**19.4**	**< 0.001**	**13.0**	**25.8**
**SPMSQ**	**− 3.8**	**< 0.001**	**− 4.4**	**− 3.2**
**ASA grading**	**− 15.6**	**< 0.001**	**− 20.2**	**− 11.0**
**Handgrip strength** (kg)	**0.94**	**< 0.001**	**0.68**	**1.2**
**Types of fracture** (PTF vs. FNF)	**− 7.0**	**0.006**	**− 12.0**	**− 2.0**
Surgical delay (hour)	− 0.001	0.81	− 0.01	0.009
Surgical classification (Joint replacement vs. ORIF)	0.29	0.91	− 4.9	5.5
Time (min)	0.001	0.97	− 0.06	0.06
Blood loss (cc)	− 0.002	0.012	− 0.024	0.02

Abbreviations: ASA, American Society of Anesthesiologists; BMD, bone mineral density; BMI, body mass index; CCI, Charlson Comorbidity Index; FNF, femoral neck fracture; Hb, hemoglobin; PTF, pertrochanteric fracture; ORIF, open reduction and internal fixation; SD, standard deviation.
